# Editorial: Disruptors on male reproduction—emerging risk factors, volume II

**DOI:** 10.3389/fendo.2023.1247971

**Published:** 2023-07-24

**Authors:** Qing Chen, Yankai Xia, Honggang Li, Rossella Cannarella

**Affiliations:** ^1^ Key Lab of Medical Protection for Electromagnetic Radiation, Ministry of Education of China, Institute of Toxicology, College of Preventive Medicine, Third Military Medical University (Army Medical University), Chongqing, China; ^2^ Key Laboratory of Modern Toxicology of Ministry of Education, School of Public Health, Nanjing Medical University, Nanjing, China; ^3^ Institute of Reproductive Health/Center of Reproductive Medicine, Huazhong University of Science and Technology, Wuhan, China; ^4^ Department of Clinical and Experimental Medicine, University of Catania, Catania, Italy; ^5^ Glickman Urology and Kidney Institute, Cleveland Clinic, Cleveland, OH, United States

**Keywords:** disruptor, environment, male infertility, decline, sperm

Couple infertility represents a significant public issue affecting the health and financial, psychological, and social aspects of childbearing-aged couples. As reported by the World Health Organization (WHO), the number of infertile couples was 48 million in 2010 (1); thus, the current prevalence could be higher. In approximately half of these couples, a male infertility factor is identified, consistently with the presence of abnormal sperm parameters, such as abnormal number, motility, and/or morphology.

Epidemiological data indicate an increase in the prevalence of male infertility globally. The latest meta-regression analysis examining the sperm parameters of healthy subjects from all over the world reported that sperm concentration and total sperm count have halved in the last 40 years, with a higher slope after the 2000s (2). There is no apparent explanation for this evidence. Although some hypotheses have been suggested, no cause-effect relationship has been demonstrated so far. In addition, some research suggests that, despite a comprehensive diagnostic workup, there is no apparent explanation for male infertility in up to 70% of cases (3, 4), although this may be an overestimated data. Furthermore, considering the association between poor sperm quality and the greater risk of hospitalization, diabetes, cardiovascular disease, morbidity, mortality (5, 6), and cancer (7), the urgent need to better understand the etiology of male infertility and its correct treatment is easily understood.

Based on these premises, we launched this Research Topic in an attempt to collect evidence on disrupting molecules, which can – at least partially, explain the abovementioned data.

Some researchers tried to find the causes of the reduction in sperm counts in environmental pollution and its increase over the decades. Arato et al. investigated the effect of nickel oxide nanoparticles in porcine prepubertal Sertoli cells *in vitro*. They found a disruption in the cell function, proven by the altered expression and secretion of Anti-Müllerian Hormone and Inhibin B, MAPK signaling pathway, and cell viability, as supported by the increase in oxidative stress, DNA damage, and apoptosis. These findings are interesting, considering that apoptosis of immature prepubertal Sertoli cells in childhood and adolescence can explain a reduced sperm count in adulthood since these cells lose the ability to proliferate after puberty (8).

Circadian rhythm has also been questioned as possibly being involved in male infertility. This represents an underestimated and scantily studied issue, deserving of more attention. An overview of the mechanisms by which abnormal circadian rhythm can disturb the hypothalamus-pituitary-gonadal axis is provided in the article by Li et al. Another topic on which there is scant information is the relationship between snoring and erectile dysfunction. Interestingly, Xiong et al. reported genetic evidence for the possible causal relationship between these two conditions.

Metabolic disorders have also been investigated in an attempt to understand their role in the downward trend of sperm count (9). Majzoub et al. focused on the impact of body composition on male sexual function and found a negative correlation between metabolic age, body weight, and fat composition with testosterone level and the International Index of Erectile Function 5-item score, in subjects younger than 40 years old. Tavlo et al. hypothesized the role of metformin as a reproductive toxicant, based on the available evidence already present on this drug, but also on its presence, documented globally, in freshwater and even drinking water.

Xiong et al. investigated the epigenetic profile of 36 patients with idiopathic non-obstructive azoospermia (NOA), reporting a dose-depended decrease in the global N6-metyladenosine (m6A) methylation, in patients with a higher degree of severity of the testicular histology (normal spermatogenesis, hypospermatogenesis, maturation arrest, and Sertoli-cell only syndrome). They also reported four downregulated genes that showed a significantly lower expression of m6A methylation.

Sperm DNA damage is a biofunctional marker of semen quality and it has recently been included in the latest WHO manual, due to the large number of data documenting its role in fertility. Sperm DNA fragmentation is the subject of two articles published in this Research Topic, both by Zhu et al.

Despite its increasing prevalence, management of male infertility is expensive for the patient, and patients often cannot afford the diagnostic and therapeutic workup required. The article by Wang et al. provides a feasibility analysis of incorporating infertility into medical insurance in China and underlies the challenges in undertaking assisted reproductive techniques from the patient’s perspective.

Finally, research is urgently needed to understand the etiology of apparently idiopathic forms of male infertility, as well as the decline in sperm counts. This Research Topic attempts to partially undertake these aspects, although a huge amount of work still needs to be done. We are thankful to the authors who submitted their valuable research to our Research Topic.

## Author contributions

All authors listed have made a substantial, direct, and intellectual contribution to the work and approved it for publication.
